# A Simple Formula of the Endophytic *Trichoderma viride,* a Case Study for the Management of *Rhizoctonia solani* on the Common Bean

**DOI:** 10.3390/life13061358

**Published:** 2023-06-09

**Authors:** Khalid M. Ghoneem, Abdulaziz A. Al-Askar, WesamEldin I. A. Saber

**Affiliations:** 1 Seed Pathology Research Department, Plant Pathology Research Institute, Agricultural Research Center, Giza 12619, Egypt; khalidghoneem@arc.sci.eg; 2 Botany and Microbiology Department, Faculty of Science, King Saud University, Riyadh 11451, Saudi Arabia; 3 Microbial Activity Unit, Microbiology Department, Soils, Water and Environment Research Institute, Agricultural Research Center, Giza 12619, Egypt

**Keywords:** bioagent, simple formula, marketable formula, molecular identification, GC-MS analysis, physiological features, growth, yield

## Abstract

The utilization of beneficial endophytic microorganisms presents a promising and innovative strategy for attaining environmental sustainability and fostering development. The majority of microbial bioagents are unsuitable for preparation in a suitable granular formula, and few are prepared in complicated formulas. In this work, *Trichoderma viride* was simply prepared in a marketable granular formula to manage *Rhizoctonia solani* and improve common bean growth. The GC-MS analysis showed several antimicrobial compounds in the fungal filtrate. *T. viride* was able to suppress the phytopathogenic *R. solani* in the laboratory. The formula had up to 6 months of shelf-life viability. Under greenhouse conditions, the formula improved plant resistance against *R. solani*. Moreover, the vegetative plant growth and physiological performance (peroxidase, polyphenol, total phenols, phenylalanine ammonia-lyase, and photosynthetic pigments) of the common bean showed obvious promotion. The formula reduced the disease incidence by 82.68% and increased the yield by 69.28%. This work may be considered a step in the right direction for producing simple bioactive products on a large scale. Moreover, the study’s findings suggest that this method can be considered a novel approach to enhancing plant growth and protection, in addition to reducing costs, improving handling and application, and maintaining fungal viability for enhancing plant growth and protecting against fungal infections.

## 1. Introduction

By utilizing beneficial microorganisms, biological control can effectively combat plant diseases by diminishing the presence of phytopathogens, and/or bolstering plant resistance [1]. Among biocontrol agents, *Trichoderma* genus has a meaningful effect on the progression of plant diseases resulting from many phytopathogenic fungi, such as *Rhizoctonia solani*, *Sclerotinia rolfsii, Verticillium dahliae,* and *Fusarium* spp., as well as oomycete-related pathogens comprising *Pythium* spp. and *Phytophthora* spp. [2,3,4,5,6,7]. *Trichoderma* species are believed to be successful biocontrol agents in soil ecosystems, due to their capacity to colonize and for their rapid growth near plant roots, their ability to utilize diverse substrates, resilience to various toxic chemicals and pesticides, and capability of modulating environmental situations [2,7]. Different direct and indirect antagonistic means have been identified. Direct ways include competition for area and nutrients, antibiosis, and mycoparasitism. The indirect ways comprise the activation of local or systemic defense responses and the improvement of plant development [2,4]. On the other hand, several metabolites of *Trichoderma* spp. that modulate plant growth such as gibberellin, indole-3-acetic acid, and auxin were also reported [2,8]. In this way, the biocontrol mechanisms are induced, including the *Trichoderma* spp. genes responsible for the expression of secondary metabolites, signaling molecules, siderophores, plant growth regulators, and the degradation of cell wall enzymes [7]. The majority of microbial bioagents cannot be prepared in a suitable formula, and few are prepared in complicated formulas.

The common bean is a valuable supply of dietary protein, which comprises 22% of its weight, and is responsible for providing protein (15%) and 30% of the caloric needs of the global population, particularly those who are underprivileged. The common bean makes up 50% of the consumed legumes around the world [9]. In addition, the seeds are recognized as a source of various antioxidants, minerals, and polyphenols [10]. Moreover, the dry common bean contains 60.6% carbohydrates, 1.3% fat, and 3.2% minerals, and vitamins (mg/100 g) such as riboflavin (0.2), thiamine (0.6), ascorbic acid (2.0), and nicotinic acid (2.5) are also present [11]. Furthermore, this plant can preserve soil fertility, due to its exceptional capacity to convert atmospheric nitrogen into a usable form, which means it can thrive without the need for highly fertile land [12].

Unfortunately, this valuable and economic crop is subjected to infection by various fungal pathogens, leading to various losses in the amount and quality of the seed yield. The common bean is attacked by the fungus *R. solani* Kühn (teleomorph: *Thanatephorus cucumeris* (A.B. Frank) Donk). It is a principal cause of web blight and root rot worldwide [13,14], leading to yield losses of up to 100% under severe field conditions [15]. In its wide host range and its survival ability, which it achieves by developing a sclerotium (survival structure), it exhibits further complicated management strategies [16,17]. Fungicides can widely manage fungal infection, but these fungicides have several known reverse impacts on health, the environment, and the economy. Therefore, safe alternatives for managing phytopathogenic fungi are urgent and very much required.

The lack of cost-effective and efficient formulations is a major obstacle to the widespread implementation of biocontrol agents against plant pathogens. Formulations play a crucial role in safeguarding biocontrol agents from environmental stress, while also providing nourishment, enhancing longevity, and augmenting resistance against factors such as rain [18]. In addition, formulation enables the controlled release of the organism and/or its metabolites from the formula.

This facilitates the replication and growth of the organism at the infection site, creating a reservoir of microorganisms and their metabolites within the natural matrix of additives. This can guide a decrease in the cost of the final formulation and provide supplementary nutrients for the product. As a result, the formulation is effective in managing diseases and enhancing seed yield [19].

In particular, the granular formulation of *Trichoderma* can be distinguished from other types of formulations, as it can be easily applied in the field, provides greater persistence and high protection against unsuitable conditions, has visible bio-inoculums on the granules, and has long viability. Additionally, it can be used easily and efficiently by farmers, so the formulation form is, in practice, more effective [20]. This kind of formulation is promising, and studies should be complemented with those in vitro to investigate its biocontrol capacity, thus facilitating its application and distribution [21,22].

The present study aimed to set up a simple procedure to generate a value-added product through the biotransformation of the endophytic *T. viride* into a bioagent formula. The shelf-life of the resulting marketable formula was evaluated before it was tested as a growth promoter as well as a bioagent and inducer of plant systemic resistance against *R. solan*. This phytopathogenic fungus was used as a model for the evaluation of the formula. A case study was performed on the prepared formula to experimentally validate its effectiveness on the common bean as a plant model.

## 2. Materials and Methods

### 2.1. The Pathogenic and Endophytic Fungi

A severe isolate of *Rhizoctonia solani* Rs77 and the endophytic *T. viride* AKW were previously isolated from common bean seeds from the Riyadh and Al-Kharj regions in the Kingdom and morphologically identified. Both fungi were sub-cultured on plates of PDA (Difco, Tucker, GA, USA), and incubated (25 ± 2 °C, 5 days).

### 2.2. Molecular Identification

Both fungi were molecularly identified. The DNA isolation and purification were accomplished using DNeasy Tissue Kits from QIAGEN, Taufkirchen, Germany, and the DNA concentration was measured by comparison with lambda DNA on a (1% *w*/*v*) agarose gel. The DNA molecules were subsequently diluted to 20 ng µL and stored at −20 °C. To perform amplification and sequencing of ITS, the PCR reaction was carried out in a working volume of 50 µL, composed of 1× buffer from Promega, 1.5 mM MgCl_2_, 1U of Taq DNA polymerase from GoTaq (Promega), 0.2 mM dNTPs, 30-picomoles of each primer (ITS5-F (5′-GGAAGTAAAAGTCGTAACAAGG-3′), and ITS4-R (5′-TCCTCCGCTTATTGATATGC-3′)), and 30 ng of DNA.

The PCR amplification was carried out using the Perkin-Elmer/GeneAmp^®^ PCR System 9700 (PE Applied Biosystems, San Francisco, CA, USA), which was adjusted to run 40 cycles (30 s each) following a start denaturation cycle at 94 °C for 5 min. The annealing stage was performed at 45 °C for 30 s, while the elongation was carried out at 72 °C for 30 s. The final cycle was extended for up to 7 min at 72 °C. For purification, the unwanted PCR segments and dNTPs from the PCR products were separated using the Montage PCR Clean-up kit from Millipore (Taufkirchen, Germany). To analyze the sequence of the nucleotide, similar nucleotide sequences at http://ncbi.nlm.nih.gov/BLAST/ (accessed on 11 December 2022) were obtained utilizing the NCBI-BLASTn. The sequences were then aligned, utilizing the BLAST with the nucleotide collection (nr/nt) database. The relationships of the taxa were compared, based on the neighbor-joining method [23]. The uncertain sites were eliminated before the evolutionary analyses [24].

### 2.3. T. viride vis R. solani

To assess the antagonistic capabilities of the *T. viride* AKW strain isolated from common bean seeds against *R. solani*, a paired culture method was employed [25]. In this method, a 5 mm diameter disc of a 5-day-old culture of *T. viride* was paired on PDA with 3 discs of *R. solani* located at the same distance from the periphery at the other side of the Petri plate. Control plates were either with the *T. viride* or *R. solani*. After incubation at 25 ± 2 °C for up to 5 days, the inward linear growth of the fungi was recorded.

The antagonistic reaction of *T. viride* was assessed, based on the interaction between the dual mycelia. The degree of antagonistic reactions was scored on a scale of 1 to 5. A score of 1 indicated *T. viride* overgrowth of the pathogen, 2 indicated that *T. viride* overgrew two-thirds of the medium surface, 3 indicated that both *T. viride* and the *R. solani* colonized one-half of the medium each, 4 indicated that the pathogen colonized two-thirds of the medium, and 5 indicated the pathogen overgrowing the *T. viride* [26].

### 2.4. Culturing Conditions

A 50 mL potato dextrose broth was autoclaved (121 °C, 15 min), injected with *T. viride* (1.0 mL of 10^8^ spores/mL), prepared from a 7-day-old colony, and incubated for 14 days under static conditions. The flask contents were put in a blender and blended. After filtering the fungal culture through filter paper, the resulting filtrate was further clarified by centrifugation at 5000 rpm for 20 min.

### 2.5. Analysis of Gas Chromatography–Mass Spectrometry (GC-MS)

The metabolite derivatization of the *T. viride* hydrolysate was performed. First, 50 µL of BSTFA was added to the filtrate and incubated (70 °C, 30 min) in a Dry Block Heater. The GC-MS analysis was conducted using Agilent Technologies equipment, including a mass spectrometer detector (5977A) and a gas chromatograph (7890B). Separation of the metabolites was carried out using an HP-5MS column (30 m × 0.25 mm) and a film width of 0.25 μm. The flow rate of the carrier gas (hydrogen) was maintained at 1.0 mL/min in a splitless mode with an injection volume of 2 µL. The initial thermal programming was set to 50 °C for 1 min, followed by a temperature ramp of 10 °C/min until reaching 300 °C, where it was held for 20 min. Both the injector and detector were maintained at 250 °C. Mass spectra were attained via electron ionization (70 eV), with a spectral range of 30–700 *m*/*z* and a solvent delay of 9 min. The mass unit was set to 230 °C and the Quad temperature at 150 °C. The data from Wiley and NIST were utilized to match the spectra of the different components.

### 2.6. Granular Formulation of T. viride into Marketable Product

A new proposed marketable formula was manufactured using the *T. viride* filtrate containing the conidia of the bioagent fungus. For manufacturing of the marketable formula, the dough was prepared by the blending and homogenization of the *T. viride* filtrate containing conidia of the fungus (1000 mL) with a suitable amount of semolina durum wheat flour and kaolin; deionized water was added as needed, until the formation of a well-mixed dough. Using a manual machine (Marcato Model Ampia 150, Padova, Italy). the dough was folded and extruded multiple times at varying roller gaps until the dough became uniform. The sheets were then extruded, without being refolded, resulting in a sheet about one millimeter thick. Afterward, the sheets were left to air-dry on aluminum foil under normal laboratory settings (33 ± 2% humidity, 23 ± 2 °C). Once completely dried, the sheets were ground using a grinder until uniformity of granules was achieved, and the size of the granules was measured using sieves of a known size (2.25 mm)**.** After manufacturing, the formula was prepared in a marketable form by packaging in air-tight plastic pages, to be ready for handling and shipping purposes.

#### 2.6.1. Evaluation of the Marketable Product

Fungus survivability in the marketable granular bioactive *T. viride* formula (GBTF) was evaluated after the manufacturing and packing processes, to ensure the viability and suitability of the product for long-term handling and during selling processes, as well as the stability of the GBTF during various storage conditions. The spore viability of the marketable product of GBTF was verified by soaking 0.1 g granules in 10 mL of distilled water and vortexing with 5-mm diameter glass beads until the granules disappeared. Serial dilutions were made, then 1 mL was plated onto a half-strength PDA medium supported with streptomycin sulfate (0.3 g/L) and chloramphenicol (0.1 g/L). After incubation at 23 ± 2 °C for 3–4 days, the number of fungal colonies was tallied using the successive dilution procedure and presented as cfu/g GBTF. The following shelf-life viability test was performed.

#### 2.6.2. Shelf-Life Viability of GBTF

The viability of the GBTF product in relation to storage under various levels of time, temperature, and water activities (a_w_) was evaluated. This quality control test was carried out directly after manufacturing, to detect any adverse effect on the GBTF during manufacturing. The storage temperature of the GBTF was set at 4 and 25 °C, representing storage conditions in the refrigerator and room temperature, respectively, at various levels of a_w_. As previously adapted [27,28], saturated solutions (LiCl to give a_w_ 0.125 at 4, and 25 °C and Mg(NO_3_)_2_·6H_2_O to give a_w_ 0.570 at 4, and 25 °C) were utilized to maintain a constant a_w_ level. Three GBTF samples were withdrawn from each treatment monthly, for up to six months, to study the shelf-life viability of the GBTF.

### 2.7. In Vivo Experiments

#### 2.7.1. The Pathogen, and Inoculum Preparation

To prepare the *R. solani* inoculum, it was first grown on PDA and then incubated (25 ± 2 °C, 5 days). Following inoculation, mycelium disks were moved to a sterile medium containing sorghum, coarse sand, and water in a ratio of 2:1:2 (*v*/*v*) and incubated (25 ± 2 °C, 14 days). At the end of this period, the inoculum was ready for use.

In the present investigation, Rhizolex-T (50% WP) was used as a chemical fungicide positive control. The recommended dose for seed dressing was applied, which involved 3 g of Rhizolex-T per kilogram of seeds.

#### 2.7.2. Greenhouse Experimental Design

Disinfected soil consisting of clay and sand at 2:1 (*v*/*v*) was put into pots (40 cm in diameter). The pots were then individually contaminated with the pathogen, which was added at 0.4% (*w*/*w*). The pot soil was thoroughly mixed with the inoculum and watered frequently with tap water until it reached near-field capacity. The pots were then left for a week to allow for the distribution of the fungus. In other pots, 0.25 g of the manufactured product of the GBTF formula was mixed with the soil, and then five sterilized common bean seeds (the Strike variety, obtained from Holland) were sown and covered with a 3 cm thick layer of soil. Pots not inoculated with the GBTF formula served as the negative control. The pots were maintained at near-field capacity by regular watering with tap water. The following treatments were applied in this study: (1) negative control I, (2) fungicide (F), and (3) GBTF formula. In the case of infection, another group that received the same previous treatments was infested with *R. solani* (P). The design of the experimental treatments was arranged in one-way randomized blocks with ten replicate pots, which were kept under greenhouse conditions. After inoculation, the soil was watered and kept moist for the first two weeks to facilitate seed germination and the successful establishment of the fungus.

#### 2.7.3. Disease Assessment

Disease progression was estimated for every treatment (15 pots each) to evaluate the formula. After 14 days, the pre-emergence damping-off was estimated as the seeds and seedling death percentage before emergence (seed rot) with respect to the primary seed number. The post-emergence damping-off was estimated as the seedling death percentage before germination (plant infection), and the ratio of plants surviving at the end of 4 months was estimated. The disease index (DI) of Rhizoctonia-root rot was evaluated twice: one week and four weeks after the pathogen inoculation. DI was determined by assessing the root damage using the Carling et al. [29] scale as follows: 0 = no damage, 1 = minor discoloration of hypocotyl, 2 = discoloration with small necrotic lesions (<1 mm in diameter) on hypocotyl, 3 = discoloration with large necrotic lesions (≥1 mm in diameter) on hypocotyl, and 4 = death of the seedling. The following Equation (1) was used:

(1) $$DI=\frac{\sum (ab)\times 100}{AK}$$

where a is the number of plants that exhibit the same degree of infection, b is the infection degree, A is the total number of tested plants, and K is the highest infection degree (in this case = 4).

#### 2.7.4. Physiological Activities of Bean Plants

At two and four weeks after sowing, five plant samples from each treatment were carefully harvested and washed to determine their total phenol content. The Folin–Ciocalteu reagent was used [30]. Polyphenol oxidase (PPO), peroxidase (POD), and phenylalanine ammonia-lyase (PAL) were measured [31,32,33]. Six weeks after sowing, the Mackinney [34] method was used to measure the photosynthetic pigments, including chlorophyll and carotenoids.

#### 2.7.5. Analysis of Growth and Yield Parameters

For each treatment, 15 plant samples were harvested carefully. The roots were washed under running water. Shoot and root lengths, fresh and dry weights, the number of leaves and branches, and leaf area were evaluated. At maturity, the productivity criteria, including the number and weight of fresh and dry pods per plant, were determined.

### 2.8. Statistical Procedure

All trials were performed at least thrice. The greenhouse experiments (10 pots/treatment, with 5 seeds each) were arranged in a one-way randomized block. To help identify the important treatments, CoStat (version 6.4, CoHort Software, Monterey, CA, USA) was utilized for performing ANOVA, then means were presented as values ± standard deviation (SD). Means were separated by the honestly significant difference (*p* ≤ 0.05) of Tukey’s test.

## 3. Results

### 3.1. Molecular Identification

**Figure 1:**
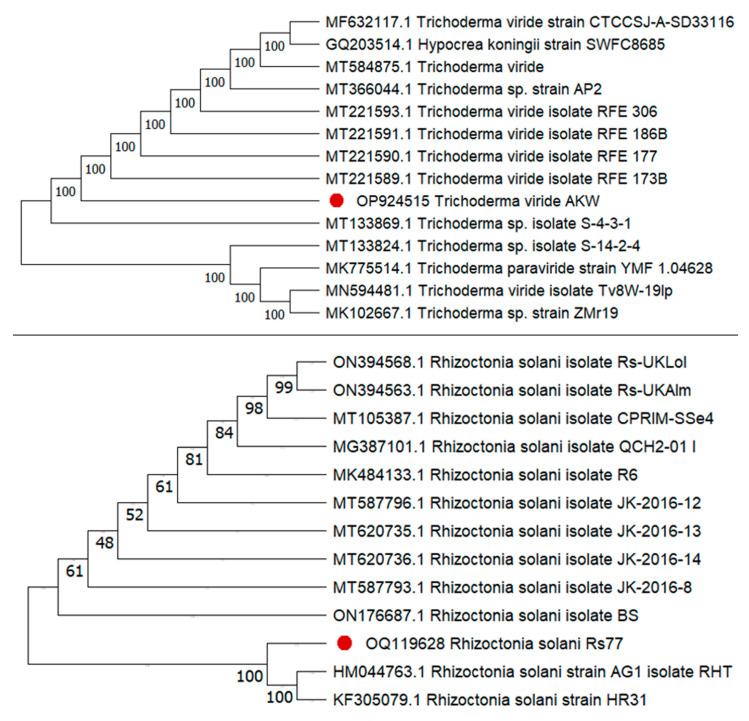
Phylogenetic tree constructed using partial sequences of the ITS, showing the location of *T. viride* AKW (MW041259) and *R. solani* (OQ119628), represented by a red dot, in relation to other related sequences attained from GenBank.

The endophytic *(T. viride* AKW), and the pathogenic (*R. solani* Rs77) fungi were molecularly identified, based on ITS (Figure 1) as a confirmed identification tool. The endophytic fungal strain, *T. viride*, displayed great similarity to the *T. viride* strains on the GenBank. The created phylogenetic tree of *T. viride* was depicted, which shows the fungus as *T. viride*. This approach aligned with the earlier identification, and the present fungal strain has been assigned to GenBank. The accession number of *T. viride* AKW was OP924515. In addition, *R. solani* Rs77 exhibited a high match with the former *R. solani* on GenBank. The created tree that was depicted came into line with the previous *R. solani* strains on the GenBank. Accordingly, the accession number was obtained as OQ119628.

### 3.2. T. viride Filtrate Analysis Using GC-MS

**Figure 2:**
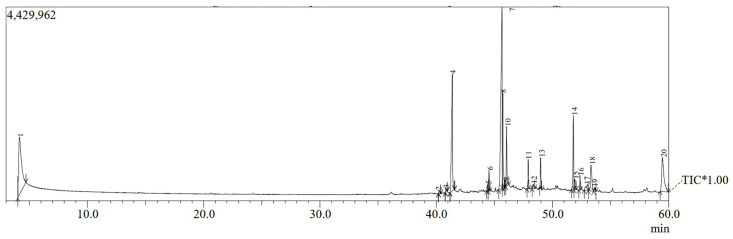
GC-MS chromatogram of bioactive metabolites of *T. viride*.

For exploring the other compounds present in the hydrolysate of the fermented plant residue, the GC-MS investigation of the cell-free culture filtrate of *T. viride* was performed, which revealed the occurrence of 20 molecules (Table 1 and Figure 2). The main constituents detected were benzenemethanol, 4-[(1-ethylpropyl)amino]-2-methyl-3,5-dinitr (37.30%), cis-vaccenic acid (21.60%), and octadecanoic acid (10.61%). Oleyl alcohol, trifluoroacetate (6.09), oleic acid (5.04%), oleoyl chloride (4.22%), octadecanoic acid (3.27% hexadecanoic acid, 2-hydroxy-1-(hydroxymethyl)ethyl ester (2.67%), palmitic acid vinyl ester (1.82%), 13-docosenoic acid, methyl ester, (Z)- (1.51%), eicosanoic acid, 2-(acetyloxy)-1-[(acetyloxy)methyl]ethyl ester (1.22%), and 9-Octadecenoic acid (Z)-, methyl ester (1.08%) were also identified, in minor amounts. The other compounds were detected at <1%.

### 3.3. Dual Culture Test

**Figure 3:**
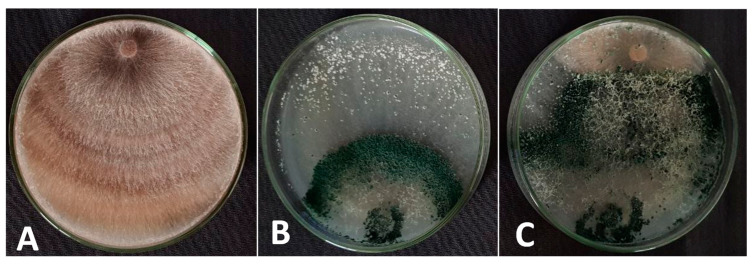
The antagonistic ability of *T. viride* AKW on *R. solani*. The radius of *R. solani* mycelium after 8 days of incubation in the control plate (**A**), the radius growth of *T. viride* (**B**), and the radius of *R. solani* and *T. viride*, showing that the bioagent covers the pathogen colony after 8 days of inoculation in dual culture plates (**C**).

A Petri plate assay was carried out to test the antagonistic properties of *T. viride* isolate against *R. solani* (Figure 3), a causal agent of root rot disease in common bean pants. The result showed a 76.80% reduction in the growth of *R. solani*. After eight days, the mycelium of the *T. viride* isolate had completely taken over the pathogen, resulting in the observation of an inhibition zone. This indicated that *T. viride* had successfully exhibited an antagonistic reaction against the target pathogen. Upon investigation using light microscopy, the hyphae appeared fragmented, vacuolated and disrupted, and there was a noticeable absence of sclerotia formation compared to the control plate. A strong mycoparasitism was also observed.

### 3.4. Shelf-Life Viability of the Marketable Formula

**Figure 4:**
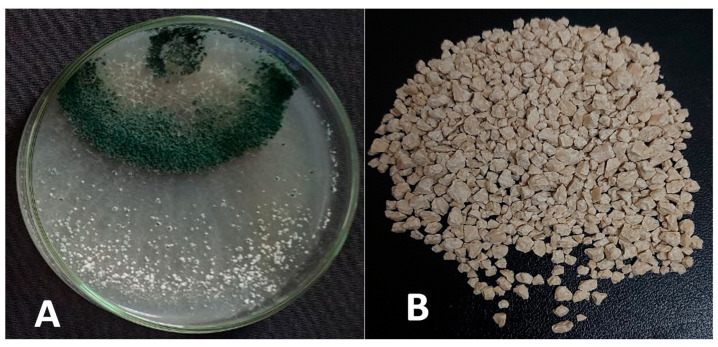
The growth of *T. viride* on Petri plates (**A**) and its granulated formula (**B**).

Before the in vivo testing of the new suggested formula, the shelf-life viability of the prepared formula (Figure 4) was studied. The data (Table 2) showed that viability sharply dropped in the stored formula at 25 °C, reaching zero after four months. The GBTF stored at a_w_ = 0.570 at 4 °C yielded the longest shelf-life and was the best GBTF when compared with the other treatments at around six months of storage, followed by a_w_ = 0.125 at 4 °C, for preserving the spore viability, compared with the control samples.

### 3.5. In Vivo Study

#### 3.5.1. GBTF Evaluation of the Development of Root Rot

**Figure 5:**
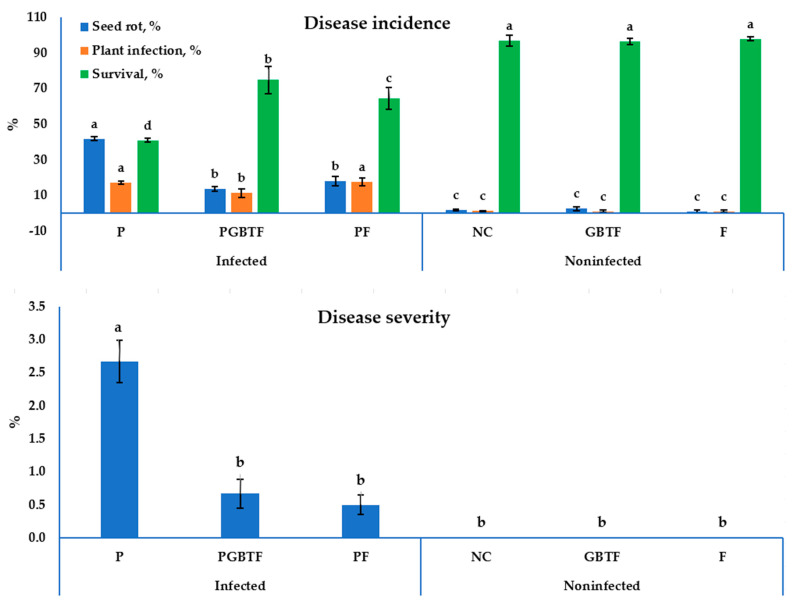
Influence of the tested GBTF treatment on the progress of disease severity, and incidence of *Rhizoctonia solani* infection of common bean under greenhouse conditions. Tukey’s test was conducted with a significance level of 0.05; the columns (means ± SD) that have a diverse letter within a parameter are considered significantly different. P; *R. solani* pathogen only, PGBF; P + PGBF, and PF; P + Rhizolex T50, NC; the negative control (no treatment), GBEF; the sole GBEF formula without infection, and F; the recommended fungicide without infection (15 pots/treatment).

*Phaseolus vulgaris* was selected as a plant model for the evaluation of the formula, since it is important to consume food legumes globally, including in developed and developing countries. Evaluation of the GBTF formula under epiphytic conditions for their action on *R. solani* (Figure 5) recovered a significant variation in the DI and DS in common bean plants. Connected with this, under infection stress by *R. solani*, the control treatment showed the highest mortality of seed and seedling, with 41.8 and 17.2%, respectively, and the survival of the common bean seedlings was reduced (41.0%); the conditions also caused the highest increase in the DI symptom, which reached 2.67. On the other hand, GBTE application resulted in a significant reduction in the *R. solani* infection, both in seeds (13.7%) and seedlings (11.40%), and consequently increased plant survival by up to 74.90%. The biotreatment with GBTE also resulted in appreciable decreases in the DS parameters, with up to 0.67 without significance with the chemical fungicide treatment (0.50) compared with the infected control (2.67).

#### 3.5.2. Growth and Yield

**Figure 6:**
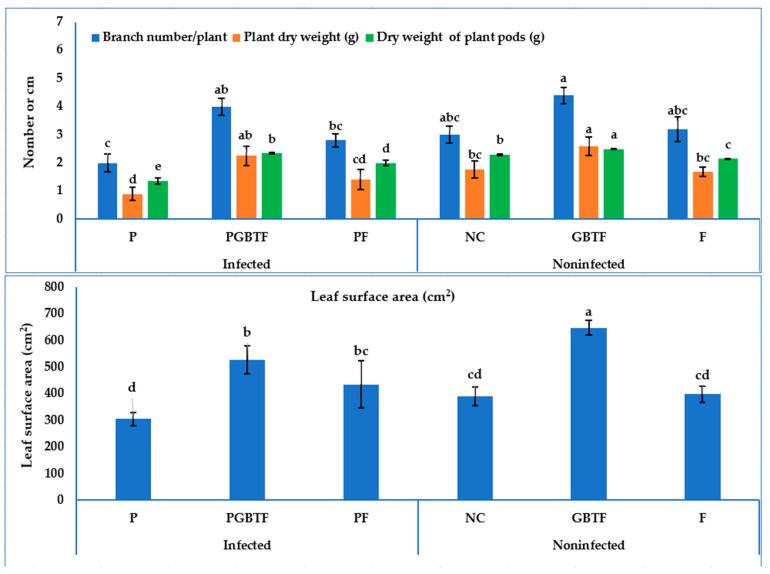
Growth and yield of common bean as affected by GBEF treatment under greenhouse conditions. Tukey’s test was conducted with a significance level of 0.05; the columns (means ± SD) that have diverse letter(s) for each criterion are considered significantly different. P; *R. solani* pathogen only, PGBF; P + PGBF, and PF; P + Rhizolex T50, NC; the negative control (no treatment), GBEF; the sole GBEF formula without infection, and F; the recommended fungicide without infection (n = 15 plant/treatment).

**Figure 7:**
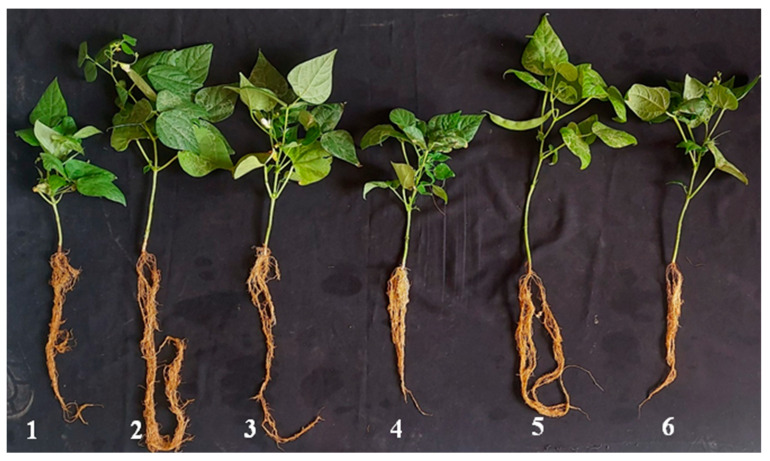
Influence of GBTF treatment on common bean growth under greenhouse conditions: (1) *R. solani* pathogen (P), (2) P + GBTF, (3) P + the fungicide (F), (4) control without any treatment, (5) the single GBTF formula without infection, and (6) the single F without infection.

Figure 6 and Figure 7 and Supplementary Table S1 display the effect of biotreatment with the GBTF formula on the growth characters of the plants. Under infection stress, data reveal that GBTF treatment was the best regarding the advancement of the shoot length, branch number, and the plant’s fresh and dry weight (27.50 cm, 4.0, 16.25 g, and 2.25 g, respectively). Fungicide treatment came after, in this respect (20.50 cm, 11.05, and 1.41 g, respectively), in comparison with the untreated-infected control (14.0 cm, 6.10, and 0.90 g, respectively). On the other hand, the biotreatment was equal to the fungicide in increasing root length, leaf number, and leaf surface area parameters of plants (32.33, 29.00 cm, 8.5, 6.83, and 527.82, 435.96, respectively), compared with the untreated-infected control (26.33 cm and 5.17, respectively). Concerning the untreated-infected control plants, the application of the GBTF formula reflected a good significant increment in the yield parameters of the plants (pod number and fresh and dry plant^−1^), with 7.33, 11.84, and 2.34 g, respectively. Compared to the untreated plants, the use of the GBTF formula led to an increase in all the tested features.

#### 3.5.3. Physiological Activities of Common Bean Plant

The physiological parameters of the common bean in terms of total phenol, PPO, POD, and PAL enzymes activity and photosynthetic pigments contents in all the investigated GBTF are displayed in Table 3 since they have a vital task to perform in plant defense. Under infection by *R. solani*, GBTF led to the highest increase in the total phenolic contents (31.62 mg/catechol 100 g^−1^ F.W.), activities of PPO (2.31 Unit. min^−1^ g^−1^) and PAL (163.0 Unit. h^−1^ g^−1^). On the other hand, there is no significance among the treatments of GBTF, fungicide, and the infected control on POD activity. Concerning the untreated plants, the application of the GBTF formula led to a significant augmentation in plant content of total phenolic compounds, POD, PPO, and PAL enzymes. The content of leaf pigments was reduced due to the pathogen. In the presence of *R. solani*, soil treated with formula and seed treated with chemical fungicide lessened the harmful impact of the pathogen, which was accompanied by significant increases in Chl a, Chl b, and total Chls: (2.525 and 2.354, 1.123 and 1.095 and 3.648 and 3.448, respectively), were recorded. Concerning carotenoids, the application of the biotreatment did not result in a significant increase in carotenoids compared to the infected control and recommended fungicide treatments. The non-infected plants showed no significant differences among the GBTF formula, the fungicide, and the negative control, in all chlorophyll contents.

## 4. Discussion

Endophytic fungi represent a portion of the plant microbiome which can promote plant growth through enhanced nutrient solubilization in the rhizosphere, altering the stress reactions of the plant via phytohormone biosynthesis, and have antagonistic capabilities of controlling phytopathogens via the secretion of antimicrobials [2,4]. The *Trichoderma* genus is classified as one of the most popular saprophytic fungi, often living as the endophyte mycobiome of several plants. The present work aimed to exploit the unique features of the endophytic fungi in the plant production sector. As a case study, the current endophytic fungus, *T. viride* AKW, was simply biotransformed into a bioagent formula. The simply prepared formula was then evaluated as a growth promoter as well as a bioagent and inducer of common bean systemic resistance against the *R. solani* pathogen.

The endophytic fungal strain (*T. viride* AKW), and the pathogenic fungus (*R. solani* Rs77) were identified using the molecular procedure. Due to its accuracy and specificity, molecular identification has broadly been utilized as an effective and fast technique of fungal classification. The ITS regions have a uniform fragment size in many fungal clusters. Sequencing the ITS regions is necessary for revealing interspecific and sometimes intraspecific variations, making it an essential step in fungal identification. As a result, the molecular procedure has been deemed a perfect means for identifying fungi [35].

The sequence of the ITS region used for creating phylogenies is thoroughly annotated and exhibits a strong relationship with related sequences in the GenBank. This non-functional region is consistent, and frequently highly divergent among different fungal species, which assists in uncovering interspecific and, in certain instances, intraspecific variations among them [36]. This ITS is easily amplifiable from a small amount of DNA due to the multi-copy feature and thus may be considered adequate for identifying fungi at the species level [35,37]. As a result, sequencing the ITS region is regarded as a fast and highly accurate method for identifying fungi. Moreover, it can be utilized for barcode recognition of a wide range of fungal groups [38].

The GC-MS profile of the selected *T. viride* AKW cell-free culture filtrate showed the presence of highly concentrated bioactive substances. In this respect, GC-MS investigation revealed the content of unsaturated fatty acids such as hexadecenoic, oleic acid, octadecanoic acid, and palmitic acid in the *T. viride* filtrate. The bioactive constituents of the *T. viride* acetonic extract detected by GC-MS were palmitic acid, oleic acid, and octadecenoic acid, while hexadecanoic acid was reported as the main active component of *T. harzianum* [39]. Several studies have reported the antibacterial and antifungal activity of free fatty acids from various organisms [40,41,42]. In this respect, Lanzuise et al. [42] reported a strong antifungal behavior of long-chain (C14-C20) fatty acids of vegetable origin plus *T. harzianum,* and *T. virens*. Palmitic acid and acetic acid were reported as the main bioactive constituents of *T. viride* and *T. harzianum*, which have high antimicrobial activity against the growth of *F. verticillioides* and *F. proliferat um* [39]. This was also confirmed by previous studies that reported the antifungal power of palmitic acid and octadecenoic acid against the growth of several fungal pathogens [43,44]. Several unsaturated fatty acids, e.g., oleic, linolenic, and erucic acids, have been reported to exhibit fungicidal activity against phytopathogenic fungi such as *R. solani,* and *Pythium ultimum* [45]. Similarly, Altieri et al. [46] reported a greater reduction in fungal growth due to butyric, caproic, lauric, myristic, caprylic, palmitic, oleic, capric, and linoleic acid treatments. Moreover, oleic acid C18.1 (63.18%), n-hexadecanoic acid (6.17%), and ethyl oleate (4.93%), with antioxidant and antibacterial activities, were detected in the *T. atroviride* strain KNUP001 [47]. Furthermore, 13-docosenoic acid, methyl ester, (Z)- was identified as a minor compound belonging to the fatty acid methyl esters. Most of the compounds in this group have antifungal activity [48,49].

Shaaban et al. [50] detected hexadecanoic acid methyl as the main active compound in clove alcoholic extract. Its highest antimicrobial effect against clinical pathogens may be due to the proteins damaging and combining with the cell-membrane lipids that alter its permeability and lead to bacterial death [51]. Cis-vaccenic acid is an omega-7 fatty acid with antibacterial activity, as well as a hypolipidemic effect in rats [52]. The chemical compound was also reported among volatile organic compounds of the methanolic extracts from *R. longibrachiatum* AU158, which have antifungal activity against the growth of *Fusarium xylarioides* [53]. Cis-vaccenic acid is an omega-7 fatty acid that was also noted in the ethyl acetate extract of endophyte isolate *Bipolaris australiensis*, which has antibacterial properties [54]. Benzenemethanol, 4-[(1-ethylpropyl)amino]-2-methyl-3,5-dinitr was reported as a major bioactive chemical compound identified in the methanolic extract of *Aspergillus terreus*, and further, has antibacterial and antifungal activities [55]. Indeed, the antifungal potency can be connected to the main chemical constituents and also to the other minor bioactive compounds. Therefore, the reported bioactivity may be owing to the collaboration effect among the various components present in the fungal filtrates [39].

In a dual culture assay, the endophytic fungal strain *T. viride* AKW showed a pronounced antagonism against *R. solani*, recording 76.80%. The high level of the antagonism reaction means strong mycoparasitism. In this respect, the bioagent was able to develop an inhibition zone and cover and sporulate over the *R. solani* colony. The results are in alignment with those of Singh et al. [56] who reported the biocontrol capability of *T. viride* and *T. harzianum* of causing negative morphological and physiological differences in *R. solani* hyphae, such as knotting, swelling, crumpling, shriveling, flattening, bursting, and cytoplasm release, as well as an increase in plant defense-related enzymes. *Trichoderma* spp. are prospective biocontrol agents against *R. solani*, through mycoparasitic, antibiosis, and/or competition as well as the stimulation of plant defense reactions, including systemic resistance or systemic acquired resistance, known as Tricoderma-ISR [7,57]. *Trichoderma* spp. primarily employ mycoparasitism as their main antagonistic mechanism. The initial phase in this process is the *Trichoderm* spp. recognizing the fungal pathogen and subsequently the adhesion to and coiling around the target hyphae, which includes recognizing the oligopeptide and oligosaccharide compounds formed by *R. solani* in reaction to hydrolytic enzymes such as proteases and chitinases [7,58].

During mycoparasitism, *Trichoderma* spp. secrete cell-wall-destroying enzymes and, potentially, antibiotics. These activities lead to parasitism and dissolution of the cell walls, constructing direct entry zones for hyphae into the pathogenic fungus [2,4,59]. Kotasthane et al. [60] indicated that *T. viride* highly antagonized the fungal pathogens through the biosynthesis of cellular-wall-degrading enzymes including chitinase, and cellulose. In this respect, the mycoparasitism of *T. viride* with *R. solani* was associated with morphological changes including hyper-parasitic coiling around the hyphae of the pathogen and the formation of appressoria-like penetrating structures. This phenomenon is based on lectin recognition from *R. solani’s* cell wall, which results in the dissolution of the cell walls [7]. Krishnamurthy et al. [61] concluded similar findings when screening the antagonistic ability of thirty-five strains of *T. viride* and *T. harzianum* against *R. solani*. As biocontrol agents or biological fungicides, the extracellular metabolites of *Trichoderma* spp. can be used to fight off plant pathogens, which involves their installation on volatile and water-soluble metabolites [2,4] and low molecular weight metabolites [60]. The above results were also approved by the work of Awad et al. [59], who recorded the antifungal activity of the *T. viride* filtrate against the growth of different pathogens, e.g., *Fusarium solani*, *R. solani,* and *Sclerotium rolfsii*. In this respect, Abbas et al. [57] reported that proteins produced by *Trichoderma* spp. play a role in mycoparasitism, and are linked with several biological processes, including the synthesis of bioactive metabolites and the completion of the genetic reprogramming of gene expression, as well as genetic recognition and signal transduction.

The signaling pathways of cAMP and MAP kinase (e.g., G-protein subunits) regulate antibiotic production, enzyme biosynthesis, and the coiling of *R. solani*. The proteinase gene (prb1) of *T. atroviride* expressed the G-gene (tga1) [62]. The overexpressing *T. viride* tga1 exhibited a greater capacity to outgrow *R. solani* [7].

Antibiosis is another biological action in which *Trichoderma* spp. antagonizes *R. solani* through the toxicity of the secondary metabolites, which is controlled by the synthesis of several genes in the bioagent [63]. In this respect, *Trichoderma* spp. genes encode secondary metabolites (e.g., polyketides, pyrones, peptaibols, gliovirin, gliotoxin, and terpenoids), which have been reported to be poisonous to *R. solani* [7].

For assessing the potentiality of the commercial use of the endophyte *T. viride*, the GBTF shelf-life was examined at two degrees of storage temperature (4 and 25 °C) and two tiers of water activity (0.125 and 0.570). The validity of the granular formulation was best preserved when kept at 4 °C, with a water activity of 0.570. Under those circumstances, the shelf-life of the formula lasted up to six months. Therefore, further enhancement should be made to extend the shelf-life of the prepared formulation. However, the shelf life of the biocontrol product is reliant on the storage temperature and the conveyors used in the formulation of the biocontrol product, which must have a maximum degree of stability, efficiency, and survival following new biotechnological practices [64]. Chaube et al. [65] reported 82% viability for the storage of a talc-based preparation of the *T. virens* spores at 5 °C in a refrigerator after 6 months, and for 3 months at 25 °C.

Storage temperature represents the determining factor of the activity of the bioagent during the storage period; the lower the temperature, the longer the shelf-life of the GBTF. In our study, storage at 4 °C and a_w_ 0.570 was the best condition for maintaining the viability of the propagules over the storage period. This may be due to a decrease in fungal metabolic activity in such circumstances, in comparison with the high temperature (25 °C); moreover, water activity at 0.570 prevents the dehydration of the fungal cell and keeps the physiological activities at a minimum, without deterioration of the viable cell when reactivated under field conditions.

The evaluation of the GBTF formula under epiphytic conditions showed a significant reduction in the percentages of *R. solani* infection both in the seeds and at the seedling stage of the bean plants. In this connection, an appreciable decrease in plant mortality and DS parameters and a consequent increase in plant survival, of up to 74.90% without significance, with the chemical fungicide treatment were recorded. *Trichoderma* spp. can suppress pathogens through antibiosis, by the secretion of bioactive metabolites including non-volatile and volatile molecules, the most significant of which are peptaibols and polyketides [2]. In this connection, several reports indicated that the *Trichoderma* spp. exude a variety of metabolites, including terpenes, gliotoxin, pyrones, gliovirin, and peptaibols, which have antimicrobial properties and can act as a potential biocontrol to phytopathogens [65,66]. Among these antibiotics, trichotoxins A and B, trichorovins, trichodecenins, and trichocellins were reported to be produced by *T. viride* [2]. In this respect, G-protein-coupled receptors are vital intracellular signaling proteins that activate the mycoparasitic attack in the *Trichoderma* spp. [5]. However, there is a direct relationship between the enhancement of vegetative growth factors and the reduction of disease severity [67].

Biotreatments with the GBTF formula showed significant development in almost all growth and yield characteristics of the bean plants over the control, either infected or uninfected with root rot *Rhizoctonia* pathogen. These results align with *Trichoderma* fungi having established effective bio-stimulation effects for the growth and development of plants [68]. This result comes from its ability to carry out mineral solubilization, including phosphorous mobilization, through the extracellular phosphatases and by increasing the plant nutrient uptake [69], through the production of plant growth regulatory materials and phytohormones such as indole-3-acetic acid derivatives and their analogous vitamins, and through enzymes leading to the stronger root and shoot growth and organic acids in the rhizosphere such as fumaric acids and gluconic, and/or citric acids that alter the soil pH [70]. In this respect, Kotasthane et al. [60] proposed that phosphate solubilization, siderophore production, and the biosynthesis of auxin may be accountable for growth regulation in various crops. Therefore, exploring and utilizing the plant growth features occurred by promoting *Trichoderma* species may help attain sustainable and eco-friendly disease management.

The activity of the *Trichoderma* spp. can cause alterations in the chemical and physical features in the rhizosphere, including changes in Na, K, and P content, electrical conductivity, and pH. These modifications are important for increasing the availability of micronutrients and insoluble compounds, as well as siderophore production, and for the biosynthesis of auxin, leading to greater nutrient uptake, a higher photosynthetic rate, and an increase in starch accumulation [60].

Regarding the physiological aspects of common bean plants such as chlorophyll content, total phenols, and defense enzymes, e.g., POD, PPO, and PAL, the GBTF formula was the most effective treatment promoting all previous parameters. In this respect, the increment in the chlorophyll a and b content was noted in all GBTF-treated plants in the greenhouse experiments. Higher chlorophyll levels lead to better plant performance. Chlorophylls have an essential role in the enhancement of the efficacy of the photosynthetic process, enhancing plant resistance against pathogens and decreasing the photophosphorylation rate occurring after infection. By increasing plant leaf area, carbon acquisition increases strongly, leading to an improvement in yield and its components [71].

The current work showed insignificant alterations in carotenoid levels compared with the fungicide and the untreated control of the infected treatments. Carotenoids were reported to perform important functions in the defense mechanism. This also includes volatile aromatic molecules that are considered to simulate growth (through phytohormones, e.g., abscisic acid and strigolactones) and inhibit pathogens [72]. Total phenols, PPO, POD, and PAL play a central role in plant protection. Phenol compounds were boosted in GBTF-treated plants, which implies a positive correlation with plant defense. The initial step response of the plant’s defense against infection is the fast accumulation of phenols at the site of infection, which prevent or limits the pathogen growth because of its action as a photoreceptor, antioxidant, and antimicrobial [73]. PPO plays a defensive role against pathogens through various mechanisms. It produces quinones, which are toxic to pathogens and can alkylate and reduce the bioavailability of cellular proteins of the pathogen. Additionally, the cross-linking of quinones with proteins or other phenolics creates a physical fence against pathogens in the cell wall. The redox cycling of quinones also generates reactive oxygen species, such as H_2_O_2_, which are essential for plant defense signaling and communication during pathogen attacks [74].

During plant infection, there was a direct reaction by plants, whereby PPO increased up to five-fold on the first day and started to decline on the sixth day, signifying the presence of pathogens in abundance in the plant system [75]. However, many factors affect POD production, such as mechanical perturbation, which may be chronic with different intensities. POD can participate in cell wall thickness, toxin production, and stimulation of secondary metabolite secretion. Therefore, it is an essential resistance mechanism of plants versus different stresses [74].

PAL plays a fundamental role in the metabolic action of phenylpropanoid compounds by catalyzing the deamination of the amino acid L-phenylalanine, resulting in the formation of ammonia and trans-cinnamic acid. The latter, in turn, acts as a stimulant for the biosynthesis of numerous phenolic compounds, which serve as precursors in the production of esters, coumarins, flavonoids, and lignin [17]. PAL enzyme production is under control during plant growth; furthermore, it is stimulated under pathogen infection processes, and under various environmental stresses such as injury and heavy metal contamination [76]. In this respect, Surekha et al. [77] reported the active role of *T. viride* in inducing levels of defense enzymes, i.e., POD, PPO, and PAL, as well as total phenols in *Vigna mungo* plants infested with *Fusarium oxysporum* and *Alternaria alternata*. Under infection stress, the application of volatile organic compounds released from the *T. viride* significantly reduced the *Sclerotium rolfsii* infection and improved the plant growth as well as increasing the enzyme activities including PAL, PPO, β-1,3-glucanase, and the chitinases content of okra, which stimulated resistance and decreased cell death [78]. Similarly, Kumar et al. [79] recorded significant increases in the defense enzyme activity of PAL and total phenols in potato leaves resulting from seed tubers treated with *T. viride*, *T. harzianum,* and their combination under an infection challenge with *Alternaria solani*. Without infection stress, Dildey et al. [80] reported an acute increase in the PAL activity of *P. vulgaris* leaf tissue resulting from seeds treated with *T. virens* TM3, compared to the control.

In this respect, Mayo et al. [17] explained the positive effects of *T. harzianum* T019 on stimulating bean plant growth and defense in the presence of *R. solani*, through its ability to induce the expression of seven plant defense-related genes and producing a higher level of ergosterol compound. In addition, Nawrocka et al. [81] reported the ability of *T. atroviride* to promote cucumber growth and induce systemic resistance against *Rhizoctonia solani*-induced disease, which was explained by the enhancing of defense enzymes, such as syringaldazine POD, guaiacol peroxidase, PAL, and PPO. Moreover, *T. atroviride*-induced resistance might depend on the accumulation of salicylic acid derivatives and volatile compounds such as Z-3-hexenol, Z-3-hexanal, and E-2-hexenal. Similarly, Behiry et al. [82] reported the ability of the tested *T. pubescens* to exhibit a significant increase in chlorophyll content and total phenolic compounds such as chlorogenic and coumaric acid in tomato plants challenged by *R. solani infection*. In this respect, *T. pubescens* elicited induced systemic resistance in all treated tomato plants through increases in the relative expression levels of PAL, CHS, and HQT defense-related genes 15 days after the inoculations. It also exhibited increasing antioxidant enzyme production (POD, superoxide dismutase, PPO, and catalase), while high MDA and H_2_O_2_ levels were observed in the infected plants.

## 5. Conclusions

Summing up, a simple novel procedure exploited the unique features of the endophytic fungus, *T. viride*, to develop a simple marketable formula to manage *R. solani* and enhance the growth of the common bean. The formula contained several antimicrobial compounds, as shown by GC-MS analysis. The granular form of the formula maintained shelf-life viability for up to 6 months, and significantly improved plant resistance to *R. solani*. Moreover, the application of the formula under greenhouse conditions promoted vegetative growth, physiological performance, and yield. The preparation of *T. viride* in a marketable formula is expected to minimize the cost and ease the handling and application, as well as maintaining the activity of the bioagent. Thus, this simple bioactive formulation of the endophytic *T. viride* could be considered an unprecedented case study and could be applied to enhancing plant growth and protection against fungal infection, as stated in the current work.

## Figures and Tables

**Table 1:** GC-MS analysis of the bioactive metabolites of the filtrate of *T. viride* AKW.

ID	Retention Time (min)	*m*/*z*	Area	Height	Peak Area, %	The Compounds in the Order of Their Elution
1	4.147	18	5,306,194	300,206	37.30	Benzenemethanol, 4-[(1-ethylpropyl)amino]-2-methyl-3,5-dinitr
2	40.251	74	112,485	18,189	0.79	Hexadecanoic acid, methyl ester
3	40.855	55	96,629	12,072	0.68	9-Hexadecenoic acid
4	41.384	43	1,509,041	228,594	10.61	Octadecanoic acid
5	-	57	-	-	-	Isopropyl linoleate
6	44.537	55	153,693	40,184	1.08	9-Octadecenoic acid (Z)-, methyl ester
7	45.663	55	3,073,127	373,559	21.60	cis-Vaccenic acid
8	45.728	55	716,596	199,111	5.04	Oleic acid
9	45.942	67	5011	1899	0.04	Oxirane, (7-octenyl)-
10	46.056	43	464,698	111,056	3.27	Octadecanoic acid
11	47.915	43	259,321	79,493	1.82	Palmitic acid vinyl ester
12	48.359	55	78,620	11,361	0.55	Oleyl Alcohol
13	48.981	55	214,637	62,761	1.51	13-Docosenoic acid, methyl ester, (Z)-
14	51.799	55	600,665	133,963	4.22	Oleoyl chloride
15	51.919	55	125,964	21,230	0.89	Oleic anhydride
16	52.362	43	173,558	36,751	1.22	Eicosanoic acid, 2-(acetyloxy)-1-[(acetyloxy)methyl]ethyl ester
17	53.016	55	59,189	11,389	0.42	1,2-15,16-Diepoxyhexadecane
18	53.318	43	380,024	46,428	2.67	Hexadecanoic acid, 2-hydroxy-1-(hydroxymethyl)ethyl ester
19	53.659	55	29,604	7329	0.21	Cyclopropaneoctanoic acid, 2-hexyl-, methyl ester
20	59.477	55	866,355	61,073	6.09	Oleyl alcohol, trifluoroacetate

**Table 2:** The influence of the storage period, level of water activity (a_w_), and temperature on the viability of GBTF.

Storage (Month)	a_w_	GBTF Viability (cfu × 10^5^)	
		4 °C	25 °C
0		6.10 ± 0.10 a	6.10 ± 0.10 a
1	Control	5.40 ± 0.10 cd	4.13 ± 0.15 cd
2		4.20 ± 0.11 fgh	3.20 ± 0.20 e
3		3.53 ± 0.38 I	2.40 ± 0.36 f
4		2.50 ± 0.10 k	1.70 ± 0.26 gh
5		2.17 ± 0.15 k	0.00 ± 0.0 j
6		1.70 ± 0.12 l	0.00 ± 0.0 j
1	0.125	5.58 ± 0.13 bc	4.62 ± 0.08 bc
2		4.58 ± 0.11 ef	3.73 ± 0.08 de
3		4.37 ± 0.08 efg	2.37 ± 0.16 f
4		4.17 ± 0.06 gh	2.05 ± 0.22 fg
5		3.83 ± 0.13 hi	1.09 ± 0.13 i
6		3.10 ± 0.10 j	0.53 ± 0.25 j
1	0.570	5.88 ± 0.03 ab	5.69 ± 0.09 a
2		5.79 ± 0.04 abc	5.04 ± 0.19 b
3		5.49 ± 0.06 bc	4.45 ± 0.11 c
4		5.06 ± 0.08 d	3.67 ± 0.09 de
5		4.65 ± 0.10 e	2.38 ± 0.24 f
6		4.02 ± 0.08 gh	1.30 ± 0.17 hi

The count of colony-forming units at the beginning of the storage (zero time) was 6.10 × 10^5^. Tukey’s test was conducted with a significance level of 0.05; the means (±SD) that have various letter(s) within a column are considered significantly different (n = 3).

**Table 3:** Physiological features of common bean as influenced by GBTF treatment under greenhouse conditions.

Treatment		Total Phenol (mg Catechol 100 g^−1^ F.W.)	Peroxidase (Uni. min. g^−1^)	Polyphenol Oxidase (Uni. min. g^−1^)	Phenylalanine Ammonia-lyase (Uni. h^−1^ g^−1^)	Photosynthetic Pigments (mg g^−1^ F.W.)			
						Cha.	Chb.	Chls.	Carotenoids
Infected	P	21.19 ± 0.22 de	0.00 ± 0.00 b	0.00 ± 0.00 c	62.30 ± 8.73 e	1.340 ± 0.15 b	0.602 ± 0.17 b	1.943 ± 0.25 b	0.166 ± 0.08 abc
	PGBTF	31.62 ± 1.27 b	4.20 ± 0.10 b	2.31 ± 0.81 bc	163.00 ± 7.94 a	2.525 ± 0.37 a	1.123 ± 0.20 a	3.648 ± 0.56 a	0.269 ± 0.01 a
	PF	26.44 ± 0.23 c	4.24 ± 0.58 b	0.00 ± 0.00 c	77.70 ± 11.50 cd	2.354 ± 0.68 a	1.095 ± 0.27 a	3.448 ± 0.55 a	0.258 ± 0.05 ab
Noninfected	NC	20.64 ± 0.51 e	0.00 ± 0.00 b	0.00 ± 0.00 c	99.00 ± 5.29 cd	1.935 ± 0.01 ab	1.043 ± 0.01 a	2.978 ± 0.02 ab	0.161 ± 0.01 bc
	GBTF	37.68 ± 1.40 a	62.27 ± 5.54 a	66.53 ± 0.00 a	124.00 ± 5.29 b	2.477 ± 0.11 a	1.304 ± 0.06 a	3.781 ± 0.16 a	0.138 ± 0.02 c
	F	24.35 ± 2.26 cd	11.49 ± 1.46 b	4.17 ± 1.83 b	101.00 ± 3.79 bc	2.252 ± 0.19 a	1.025 ± 0.09 a	3.277 ± 0.29 a	0.221 ± 0.02 abc

Tukey’s test was conducted with a significance level of 0.05; the means (±SD) that have a diverse letter(s) within a column are considered significantly different. P; *R. solani* pathogen only, PGBF; P + PGBF, and PF; P + Rhizolex T50, NC; the negative control (no treatment), GBEF; the sole GBEF formula without infection, and F; the recommended fungicide without infection (n = 5 plants/treatment).

## Data Availability

All data were reported in the paper and the Supplementary Materials.

## Supplementary Materials

The following supporting information can be downloaded at https://www.mdpi.com/article/10.3390/life13061358/s1, Supplementary Table S1. Growth and yield of common bean as affected by GBEF treatment under greenhouse conditions.
