# Supplementary material for: A Simple Formula of the Endophytic Trichoderma viride, a Case Study for the Management of Rhizoctonia solani on the Common Bean
Source: Life (Basel). 2023 Jun 9;13(6):1358. doi: 10.3390/life13061358 (PMC10300855; doi:10.3390/life13061358)
Supplement: Supplementary file 1 [file life-13-01358-s001.zip › life-2378288-supplementary.pdf]

A simple formula of the Endophytic *Trichoderma viride,* a Case Study for the Management of *Rhizoctonia solani* on Common Bean

Khalid M. Ghoneem [1], Abdulaziz A. Al-Askar [2,*] and WesamEldin I. A. Saber [3,*]

Supplementary Table S1. Growth and yield of common bean as affected by GBEF treatment under greenhouse conditions.

Treatment		Plant length (cm)		Leaves Number plant^{-1}	Plant fresh weight (g)	Pods per plant^{-1}	
		**Shoot**	**Root**			**Number**	**Fresh weight (g)**
Infected	**P**	14.00±2.32 c	26.33±2.17 c	5.17±0.75 b	6.10±1.61 d	4.33±0.82 b	5.68±0.55 e
	**PGBTF**	27.50±3.44 a	32.33±6.54 b	8.50±2.72 ab	16.25±1.61 ab	7.33±0.81 a	11.84±0.12 b
	**PF**	20.50±2.02 bc	29.00±4.40 bc	6.83±2.01 ab	11.05±2.55 c	5.17±0.75 b	9.97±0.13 d
Noninfected	**NC**	26.83±3.86 ab	30.25±4.78 bc	8.33±1.36 ab	12.39±2.40 bc	6.83±0.75 a	10.45±0.16 c
	**GBTF**	31.42±3.35 a	38.08±3.14 a	9.33±0.52 a	17.29±1.30 a	8.171.17± a	12.39±0.11 a
	**F**	27.42±2.65 ab	31.42±2.11 bc	8.67±1.75 ab	12.77±1.16 bc	7.00±0.89 a	10.59±0.14 c

Tukey's test was conducted with a significance level of 0.05, the means (±SD) that have a diverse letter(s) for each criterion are considered significantly different. P; *R. solani* pathogen only, PGBF; P + PGBF, and PF; P + Rhizolex T50, NC; the negative control (no treatment), GBEF; the sole GBEF formula without infection, and F; the recommended fungicide without infection (n = 15 plant/treatment).
